# Trends in disability burden of site-specific osteoarthritis among elderly adults in China from 1990 to 2023 and projections to 2035: a comprehensive analysis based on GBD 2023

**DOI:** 10.1515/med-2026-1519

**Published:** 2026-11-12

**Authors:** Yuwen Wang, Longtai Li, Xiaowen Hu, Liguo Li

**Affiliations:** Institute of Rehabilitation Medicine, Henan Academy of Innovations in Medical Science, Zhengzhou City, Henan Province, China

**Keywords:** elderly adults, China, osteoarthritis, years lived with disability, projection analysis

## Abstract

**Objectives:**

The burden of osteoarthritis (OA) among elderly adults in China is increasing, yet trends for site-specific OA in this population remain inadequately studied. To explore the disease burden of OA at different anatomical sites among elderly adults in China, as well as its gender and age disparities, and to forecast future trends.

**Methods:**

Using the Global Burden of Disease (GBD) 2023 database, we analyzed the trends of hand, knee, hip, and other-site OA among Chinese elderly adults (aged ≥60 years), stratified by gender and age groups, based on the absolute number of Years Lived with Disability (YLDs) and Age-Standardized YLDs Rate (ASYR) from 1990 to 2023. Joinpoint regression and age-period-cohort models were adopted for statistical analyses.

**Results:**

From 1990 to 2023, the YLD burden of site-specific OA exhibited an upward trend among Chinese elderly adults. Hand OA presented the largest increases in total YLD count and ASYR, with growth rates of 404.45 % and 60.17 %, respectively. Knee OA imposed the heaviest disease burden (ASYR_2023_=685.02/100,000), and females bore a higher burden than males. Age-period-cohort analyses revealed distinct site- and gender-specific disparities in age, period, and cohort effects across all OA subtypes. Projections for 2024 to 2035 indicated consistent rises in total YLD numbers for OA at all anatomical sites, while the magnitude of changes in ASYR remained modest and varied by lesion location.

**Conclusions:**

Between 1990 and 2023, the overall YLD burden of site-specific OA increased among Chinese elderly adults. Hand OA saw the most substantial growth, while knee OA carried the heaviest burden, with gender and age disparities observed for OA at all anatomical sites. Future research should conduct individual-level studies based on Chinese domestic cohorts, disease registries or hospital databases. OA screening, weight control, joint protection, and health education should be strengthened in primary care and community chronic disease management programs.

## Introduction

Osteoarthritis (OA) is a progressive joint disorder caused by multiple factors [1]. Its core pathological features include articular cartilage degeneration and damage, alongside osteophyte proliferation at joint margins, which ultimately manifests as joint pain, stiffness, and dysfunction [2]. Globally in 2020, approximately 595 million people were affected by OA, accounting for 7.6 % of the population. Among individuals aged 70 and older, OA ranked as the seventh leading cause of years lived with disability (YLDs), highlighting its pronounced non-fatal burden in the elderly population [3]. However, current clinical management strategies for OA, primarily pharmacological and surgical interventions, focus on symptom alleviation and cannot achieve a cure, while potentially causing adverse effects [4]. Therefore, systematically investigating OA disease burden trends and formulating prevention policies to enhance early diagnosis and intervention are imperative for mitigating its overall impact.

China faces an extraordinarily heavy OA burden driven by its huge population and rapid population aging. The Global Burden of Disease (GBD) 2017 study indicated that OA was the 24^th^ most common cause of YLDs in China, accounting for 1.08 % of all YLDs, with YLD rates increasing with age [5]. According to GBD 2019, China had the highest number of prevalent OA cases globally (133 million) [6]. Importantly, OA is not a homogeneous condition; its epidemiological characteristics, risk factors, and impact on quality of life vary depending on the affected joint site (e.g., knee, hip, hand) [7], 8]. Consequently, investigating the disease burden by specific joint site is essential for developing precise prevention and treatment strategies. A previous study has analyzed trends in the burden of site-specific OA in China, identifying knee OA as the predominant contributor to the increasing burden [9]. However, comprehensive analyses of the high-risk elderly population remain limited, constraining the development of individualized strategies for this group.

Therefore, based on the GBD 2023 database, this study comprehensively analyzed the temporal trends and distribution characteristics of the disease burden of site-specific OA among Chinese elderly adults from 1990 to 2023, and predicted its future changes from 2024 to 2035. The findings aim to provide supplementary evidence for developing targeted, site-, gender-, and age-specific prevention and control strategies for the elderly population.

## Methods

### Data source

All data in this investigation were sourced from the GBD 2023 database (https://vizhub.healthdata.org/gbd-results/, accessed on July, 2026) using the GBD results tool. The GBD 2023 database contains comprehensive burden of disease data covering 375 diseases and injuries, along with 88 risk factors, across 204 countries and territories from 1990 to 2023.

The following data were downloaded for secondary analysis: the number of YLDs and CYR for hand, knee, hip, and other site OA among elderly adults (≥60 years) in China from 1990 to 2021, stratified by age group. Age was categorized into 5-year intervals, starting from 60 years and above. Both the counts and rates data included the estimated values and their corresponding 95 % uncertainty intervals (95 % UI).

The age-standardized rate (ASR) and its 95 % UI were calculated using the direct standardization method. The formula is as follows:

$$\text{ASR}=\frac{\sum_{i=1}^{A}a_{i}w_{i}}{\sum_{i=1}^{A}w_{i}}\times 100,000$$

*a*_*i*_ represents the crude rate in the i-th age group, *w*_*i*_ denotes the weight of the i-th age group in the standard population, and A is the number of age groups. Standardized population by age group was derived from the world standard population database [10], 11] developed by the World Health Organization [12].

### Definition of OA

OA was defined by the corresponding International Classification of Diseases (ICD) codes for specific joint sites. Within the GBD database, OA is categorized into the following types: hip OA (ICD-10: M16.0, M16.1, M16.2, M16.3, M16.4, M16.5, M16.6, M16.7, M16.9), knee OA (ICD-10: M17.0, M17.1, M17.2, M17.3, M17.4, M17.5, M17.9), hand OA (M18.0, M18.1, M18.2, M18.3, M18.4, M18.5, M18.9), and OA of other sites (M19.0, M19.2, M19.8, M19.9, M19.91, M19.92, M19.93) [6].

### Statistical analysis

#### Descriptive analysis

This analysis delineated the temporal trends in YLDs burdens attributable to site-specific OA among Chinese elderly adults, stratified by gender (males and females), from 1990 to 2023. It also quantified disparities in YLD counts across gender and age subgroups in 2023.

#### Joinpoint regression analysis

We introduced Joinpoint Regression Program (version 5.2.0) [13] to analyze the temporal trend. A segmented linear model on a logarithmic scale was fitted to the annual rates, with potential inflection points identified via Monte Carlo permutation tests (overall significance level α=0.05, 0 to 5 candidate joinpoints). The trend within each segment was expressed as the annual percent change (APC). To facilitate an overall comparison, the average annual percent change (AAPC) along with its 95 % Confidence Interval (CI) was calculated. An upward trend was defined when the lower limit of the 95 % CI for the APC or AAPC >0, a downward trend when the upper limit <0, and a stable trend when the 95 % CI included 0.

#### APCM

The APCM was employed to disentangle the driving factors behind changes in the disease burden by identifying age, period, and cohort effects: the age effect reflects inherent differences across age groups, the period effect captures external factors influencing all age groups simultaneously at a given time, and the cohort effect stems from the variance in early-life experiences of different birth cohorts. The basic form of the model is:

$$\log\;Y_{\text{ijk}}=\mu +\alpha_{i}+\pi_{j}+\gamma_{k}+\epsilon_{\text{ijk}}$$

Y_ijk_ represents the rate of the disease burden indicator, μ is the intercept, αᵢ is the age effect, πⱼ is the period effect, γ_k_ is the cohort effect, εᵢⱼ_k_ is the random error term. The model suffers from the identifiability problem due to the linear dependency (Cohort=Period – Age), which was addressed by the intrinsic estimator method [14].

Chinese elderly adults were categorized into 5-year age groups (totaling 8 groups). The study period from 1992 to 2023 was divided into 5-year intervals (totaling 6 periods). The birth cohorts included individuals born from 1899 to 1963 (13 cohorts in total). Age-period-cohort analyses were conducted separately by gender on the CYR for site-specific OA among Chinese elderly adults across these age groups. R software (version 4.3.0) was introduced for all analyses, with a p-value <0.05 considered statistically significant [15], [16], [17].

#### Projection analysis

The BAPCM was used to forecast the disease burden from 2024 to 2035. In this model, the age, period, and cohort effects were treated as random effects and assigned a second-order random walk (RW2) smoothness before balance trend capture and overfitting avoidance. Poisson regression was applied to fit the Lexis array data. Parameters were inferred by the Integrated Nested Laplace Approximation (INLA) method, yielding the posterior mean and its 95 % posterior CI as output. All analyses were conducted in R (v4.3.0) utilizing the “BAPC” and “INLA” packages.

## Results

### Temporal trends in the burden of site-specific OA among elderly adults in China, 1990-2023

Both the total YLD counts and ASYR of site-specific OA demonstrated increasing trends among Chinese elderly adults from 1990 to 2023 (Table 1, Table S1). Hand OA exhibited the largest growth in total YLD years, rising from 161,092 to 812,629, representing an increment of 404.45 %. Hip OA ranked second with a growth rate of 314.71 %, whereas knee OA had the mildest increase at 216.66 %.

**Table 1:** Changes in the burden of YLDs due to site-specific OA among elderly adults in China, 1990 and 2023.

Cause	Number			ASYR		
	1990 (95 % UI)	2023 (95 % UI)	Percentage change, %	1990 (95 % UI)	2023 (95 % UI)	Percentage change, %
Hand osteoarthritis	161,092 (70,147–352,138)	812,629 (361,505–1,792,935)	404.45	172.46 (75.78–375.42)	276.21 (123.18–608.14)	60.17
Hip osteoarthritis	28,646 (13,054–61,590)	118,797 (53,227–253,340)	314.71	30.50 (13.89–65.32)	40.53 (18.19–86.39)	32.87
Knee osteoarthritis	640,411 (304,080–1,387,003)	2,027,903 (955,642–4,409,304)	216.66	641.87 (305.55–1,388.34)	685.02 (322.97–1,488.37)	6.72
Other osteoarthritis	109,855 (49,190–238,109)	359,179 (163,146–776,443)	226.96	115.27 (52.34–248.87)	121.97 (55.48–263.34)	5.81

YLDs, years lived with disability; ASYR, age-standardized YLDs rate; UI, uncertainty interval. The unit for all rates is per 100,000 population.

In terms of ASYR, hand OA still had the highest growth rate (60.17 %), followed by hip OA (32.87 %) and knee OA (6.72 %); OA affecting other anatomical sites showed the smallest rise (5.81 %). Moreover, knee OA yielded the maximum total YLD counts and ASYR values in both 1990 and 2023, reaching 2,027,903 YLD years and 685.02/100,000 population, respectively, in 2023 (Table 1, Table S1).

### Joinpoint regression analysis of the burden of site-specific OA among elderly adults in China, 1990–2023

From 1990 to 2023, the ASYR of OA in the hands, hip joints, knee joints, and other sites among elderly adults in China all increased, with corresponding AAPC of 1.50 (p<0.001), 0.87 (p<0.001), 0.20 (p<0.001), and 0.17 (p<0.001), respectively (Table S2).

Joinpoint regression analysis revealed that knee OA consistently imposed the highest ASYR burden from 1990 to 2023, with the steepest growth observed during 2000–2005 (APC_2000-2005_=2.59). Hand OA exhibited the most prominent overall upward trend, peaking in magnitude between 2015 and 2020 (APC_2015-2020_=5.20). OA at other anatomical sites showed sustained increasing trends across all five inflection points, whereas hip OA maintained the lowest disease burden throughout the study period (Figure 1, Table S2).

**Figure 1:**
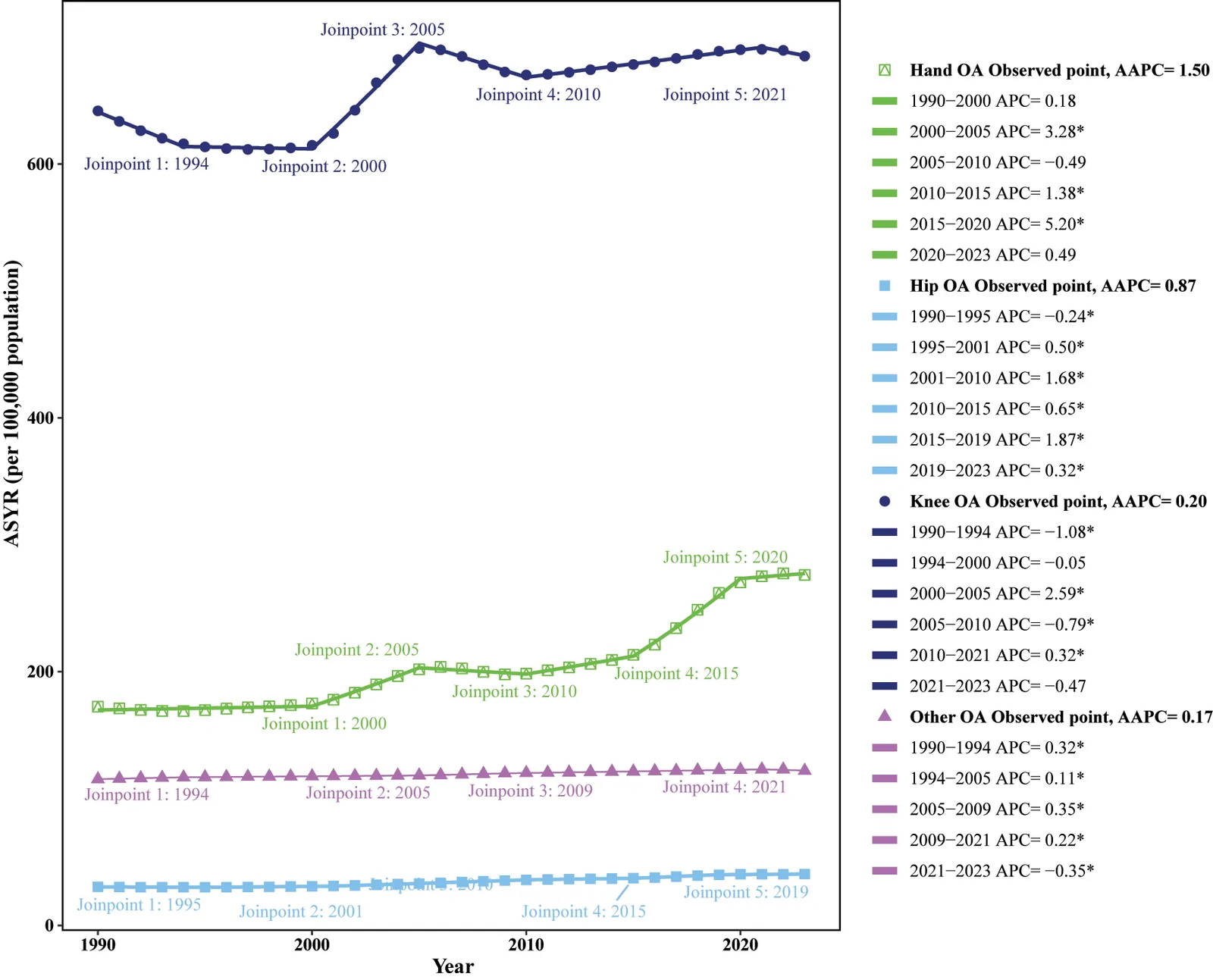
Joinpoint regression analysis of the ASYR for site-specific OA among elderly adults in China, 1990–2023. OA, osteoarthritis; ASYR, age-standardized years lived with disability rate; YLDs, years lived with disability; APC, annual percent change; AAPC, average annual percent change. *: p <0.05. Green represents hand OA, light blue represents hip OA, dark blue represents knee OA, and purple represents OA at other sites.

### Temporal trends in the burden of site-specific OA among elderly adults in China by gender, 1990–2023

The overall YLD burden of site-specific OA rose among both female and male elderly adults in China from 1990 to 2023 (Table 2, Table S3, Figure S1). Specifically, the ASYR burden of knee OA was consistently higher in females than males, while males sustained a heavier burden of hip OA and OA at other anatomical sites throughout the period (Figure S1).

**Table 2:** Changes in the burden of YLDs due to site-specific OA among Chinese elderly adults by gender, 1990 and 2023.

Sex	Cause	Number			ASYR		
		1990 (95 % UI)	2023 (95 % UI)	Percentage change, %	1990 (95 % UI)	2023 (95 % UI)	Percentage change, %
Female	Hand osteoarthritis	94,045 (40,981–206,523)	469,000 (209,164–1,048,639)	398.70	187.49 (82.35–409.89)	303.26 (135.34–677.85)	61.75
	Hip osteoarthritis	13,727 (6,253–29,608)	55,810 (25,043–119,113)	306.57	27.46 (12.51–58.98)	36.10 (16.22–77.03)	31.48
	Knee osteoarthritis	418,462 (199,094–901,258)	1,323,520 (625,585–2,866,477)	216.28	793.05 (377.97–1706.71)	857.12 (405.07–1855.61)	8.08
	Other osteoarthritis	55,652 (25,214–120,297)	177,439 (80,884–381,717)	218.84	109.88 (50.23–236.70)	114.61 (52.24–246.35)	4.30
Male	Hand osteoarthritis	67,047 (28,998–146,220)	343,628 (151,895–749,120)	412.52	153.65 (67.03–334.79)	245.99 (109.09–535.01)	60.10
	Hip osteoarthritis	14,919 (6,775–31,871)	62,988 (28,390–134,542)	322.19	34.35 (15.55–73.30)	45.52 (20.54–97.18)	32.51
	Knee osteoarthritis	221,949 (105,196–485,895)	704,383 (330,901–1,540,276)	217.36	463.98 (220.41–1,014.18)	496.72 (233.66–1,084.53)	7.06
	Other osteoarthritis	54,203 (24,042–117,493)	181,740 (82,009–393,081)	235.29	121.69 (54.96–262.66)	130.06 (58.87–280.74)	6.88

YLDs, years lived with disability; ASYR, age-standardized YLDs rate; UI. uncertainty interval. The unit for all rates is per 100,000 population.

The ASYR of hand OA exhibited divergent temporal trends between females and males, with two crossing points identified in 1992 and 2007. The female burden exceeded that of males from 1990 to 1991; males carried a greater burden between 1992 and 2006; and females gradually presented a higher ASYR burden from 2007 to 2023 (Figure S1, Table S3).

### Comparison of the burden of site-specific OA among Chinese elderly adults by gender and age group in 2023

In 2023, the YLD counts of hand, hip, knee, and other-site OA among Chinese elderly adults of both genders were predominantly concentrated in the 60–74 age group, all peaking within the 65–69 age bracket and gradually declining with advancing age after 75 years old (Figure 2).

**Figure 2:**
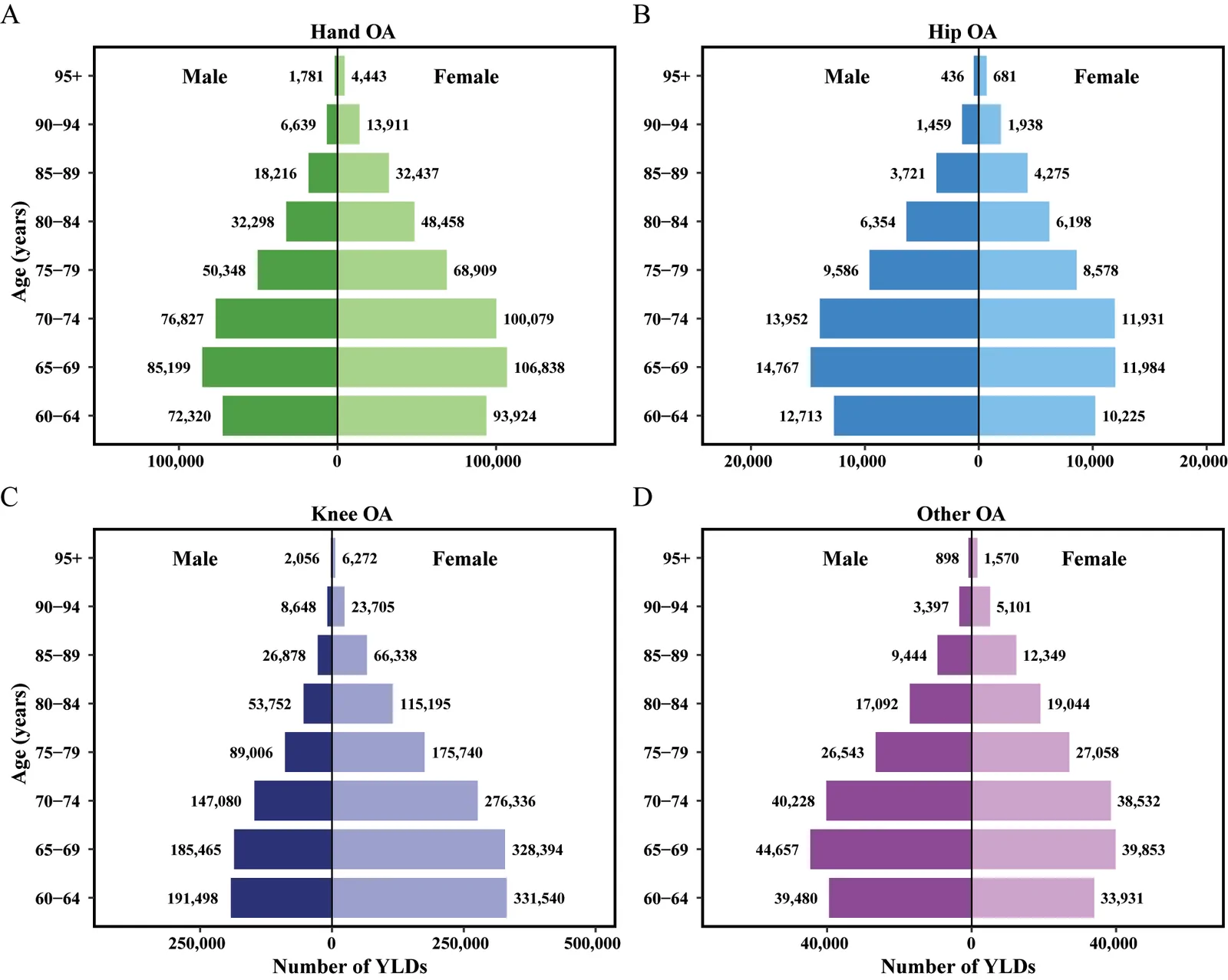
Comparison of the number of YLDs for site-specific OA among elderly adults in China by gender and age group, 2023. OA, osteoarthritis; YLDs, years lived with disability. Green represents hand OA, light blue represents hip OA, dark blue represents knee OA, and purple represents OA at other sites. Light shades represent females; dark shades represent males.

In terms of gender disparities, the YLD counts for hand and knee OA were consistently higher in females than males across all age strata in 2023 (Figure 2A and C). For hip OA and other-site OA, male YLD counts surpassed female counterparts in the 60–79 and 60–74 age groups, respectively; thereafter, female YLD counts exceeded those of males as age increased (Figure 2B and D).

### Age-period-cohort analysis of the burden of site-specific OA among elderly adults in China

Age effect analysis showed that the CYR of hand OA increased with advancing age among both male and female elderly adults in China, reaching the maximum value in the population aged 95 years and above (Figure S2, Table S4). By contrast, the CYR of knee OA rose first and then declined in both genders, peaking in the 80–84 age group (Figure S2, Table S4).

Period effect analysis revealed that for hand OA, the CYR of males in all age groups followed an up-down-up trajectory and hit its highest level during 2019–2023 (Figure S3, Table S4); the CYR of females across all age groups climbed continuously over successive periods (Figure S3, Table S4). Regarding knee OA, the CYR of all age groups in both genders exhibited a fluctuating upward trend, with peaks recorded in 2019–2023 (Figure S3, Table S4).

Cohort effect analysis indicated that for hand OA, the CYR of males and females in all age groups generally increased with later birth cohorts, with more drastic fluctuations observed in males (Figure S4, Table S5). For knee OA in both sexes, the CYR of all age groups showed a fluctuating rising trend alongside the progression of birth cohorts (Figure S4, Table S5).

### Projected trends in the burden of site-specific OA among elderly adults in China, 2024–2035

BAPC projection demonstrated that the YLD counts of site-specific OA among Chinese elderly adults would present an upward trend from 2024 to 2035. The ASYR would only rise for hip OA, while knee OA would remain the leading contributor to disease burden (Figure 3, Table S6).

**Figure 3:**
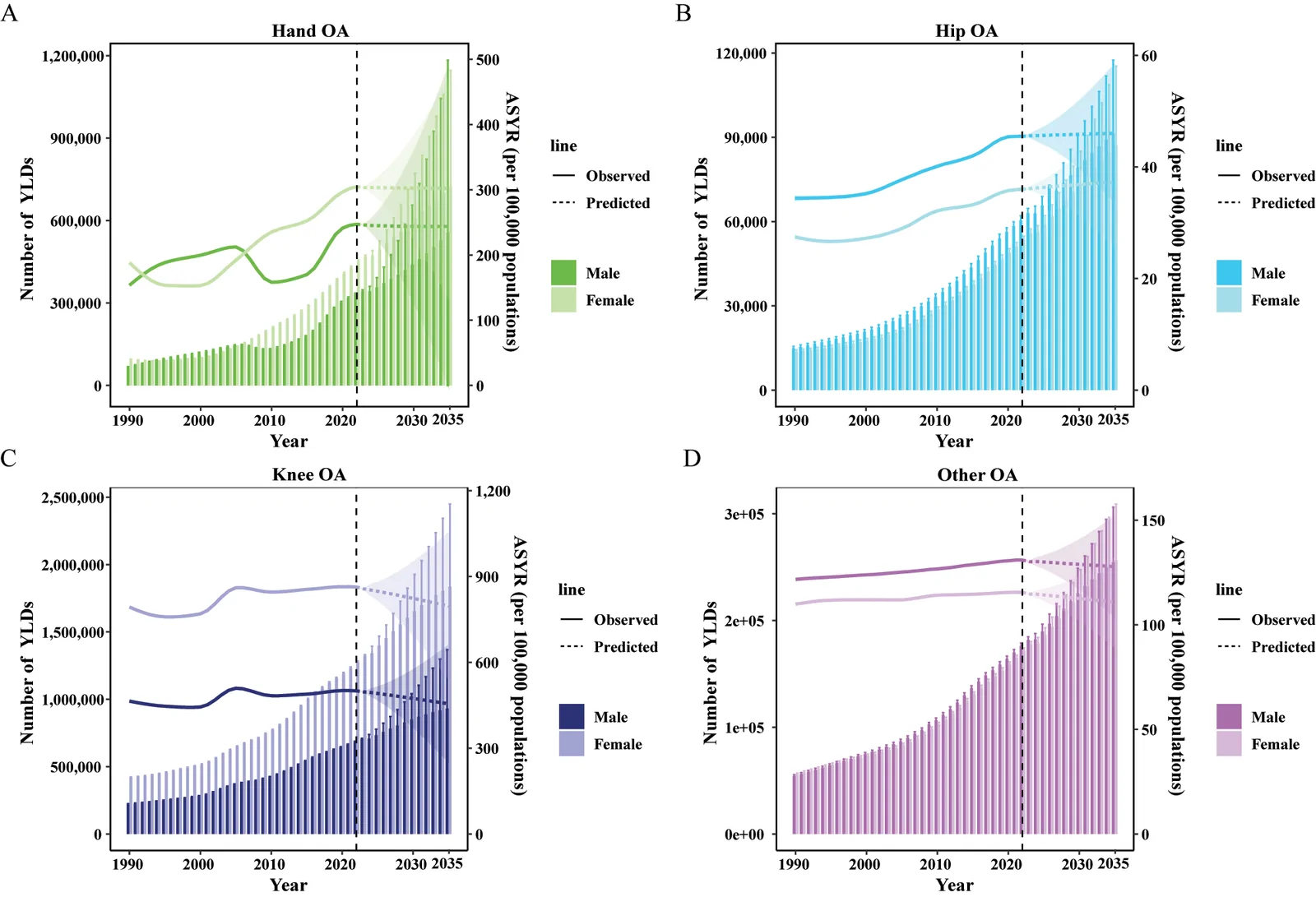
Projected trends in the number of YLDs and ASYR for site-specific OA among elderly adults in China by gender, 2024–2035, based on the BAPCM. (A) Hand OA; (B) hip OA; (C) Knee OA; (D) other OA. OA, osteoarthritis; ASYR, age-standardized YLDs rate; YLDs, years lived with disability.

From 2024 to 2035, females would consistently bear a higher YLD count burden of hand OA than males, with YLD counts increasing by 54.16 % and 61.91 %, respectively, accompanied by ASYR declines of −0.41 % and −0.92 % (Figure 3A, Table S6). For hip OA, males would sustain higher YLD counts than females, with respective growth rates of 45.08% and 55.99 %, and ASYR increments of 0.99 % vs. 3.32 % (Figure 3B, Table S6). The YLD counts of knee OA would keep rising; the YLD counts were projected to grow from 1,323,572 years in females and 704,423 years in males to 1,836,302 years in females and 930,937 years in males, representing increases of 38.74 % and 32.16 %, respectively. Nevertheless, slight ASYR reductions would be observed (females: −7.06 %, males: −8.37 %) (Figure 3C, Table S6). The YLD counts of other-site OA would rise by 44.50 % in females and 39.96 % in males, alongside ASYR decreases of −3.35 % and −1.80 %, respectively (Figure 3D, Table S6).

## Discussion

Our study revealed that the YLD burden of site-specific OA among Chinese elderly adults showed an increasing trend, with hand OA exhibiting the largest percentage increase and knee OA carrying the highest absolute burden. Significant gender-specific variations were observed for all OA sites. Projections indicates that the YLDs burden for site-specific OA is expected to continue rising in the future.

The observed increase in the YLDs burden of OA from 1990 to 2023 can be largely attributed to population aging and the obesity epidemic. According to the Seventh National Population Census of China in 2020, the population aged 60 years and older reached 264.02 million, accounting for 18.70 % of the total population [18]. The proportion of the population aged 65 and older was 13.50 %, an increase of approximately 6.5 percentage points from 2,000 (7 %), representing a near doubling [19]. Concurrently, obesity rates in China surged, with approximately 402 million adults being overweight or obese in 2021, making China the country with the highest number of overweight or obese individuals worldwide [20]. Obesity is a significant risk factor for OA, leading to increased mechanical load on joints and exacerbating cartilage wear, thereby accelerating the onset and progression of OA [21]. Furthermore, metabolic disorders and chronic low-grade inflammation associated with obesity interact with age-related declines in physical function [22], potentially worsening OA severity and contributing to the increased YLDs burden in elderly adults. Therefore, managing obesity in the elderly is an urgent priority.

Joinpoint regression analysis further pointed out that hand OA showed the most substantial increase in YLDs burden over the past three decades. This may be linked to the widespread adoption of the internet. Since the beginning of the 21st century (around 2,000), internet usage in China expanded rapidly, with elderly adults increasingly utilizing digital platforms for social interaction and entertainment [23]. The period from 2015 to 2020 witnessed the most rapid increase in the YLDs burden of hand OA. This phase coincided with the comprehensive coverage of 4G networks in China and the rapid proliferation of smartphones, which became integral to the daily lives of elderly adults for recreational purposes [24]. Behaviors such as frequent screen tapping and prolonged smartphone holding may subject the joints of the hand to excessive and biomechanically suboptimal loads, potentially leading to pain and accelerating degenerative changes [25], 26], thereby driving the increase in the YLDs burden of hand OA.

During this period, the burden of knee OA remained the highest among elderly adults in China. As previously mentioned, China experienced a sharp rise in obesity. The knee is the largest and primary weight-bearing joint in the human body; for every 1 kg increase in body weight, the force exerted on the knee joint during walking increases by 3–4 kg, accelerating the wear and tear of knee cartilage [27]. Furthermore, knee pain and functional limitations directly lead to walking difficulties, with severe cases resulting in loss of independent mobility and a sharp decline in quality of life [28], consequently contributing to a heavier YLDs burden.

Studies using publicly available domestic Chinese databases also provide qualitative references for the above findings. A national population-based study drawing on 2011–2012 data from the China Health and Retirement Longitudinal Study (CHARLS) enrolled 17,128 middle-aged and elderly participants and reported a prevalence of symptomatic knee OA of 8.1 % in China; prevalence was higher in females than males (10.3 % vs. 5.7 %) and increased with age (p for trend <0.01) [29]. These observations are broadly consistent with our finding that knee OA constitutes a heavy disease burden, with females carrying a greater burden of knee OA. Importantly, CHARLS and the present study differ incompletely from the GBD database in terms of data sources, outcome definitions, age ranges, time spans, and disease burden metrics. Accordingly, CHARLS results can only serve as a qualitative external reference and cannot be applied to validate GBD-based estimates.

Regarding gender differences, the burden of knee OA among elderly adults in China was consistently higher in females than in males from 1990 to 2023. Hormonal factors are key contributors. After menopause, women experience a sharp decline in estrogen levels and lose the protective effect of estrogen, which accelerates cartilage degeneration and a more pronounced inflammatory response [30], resulting in a greater burden from knee OA. Additionally, previous studies have reported that the YLDs rate for knee OA attributable to high body mass index was higher in women than in men [31], indicating that obesity has a more substantial impact on knee OA in elderly women. Conversely, the YLDs burden of hip OA and OA at other sites was consistently higher in men than in women. This disparity may be attributed to the higher proportion of men engaged in heavy physical labor (e.g., construction, loading/moving, mining, agriculture) compared to women [32], 33]. These occupations often involve repetitive lifting, bending, and twisting, placing substantial and sustained mechanical stress on the hip joint and other sites (such as the spine), thereby accelerating articular cartilage degeneration [34].

The trend in the YLDs burden of hand OA exhibited two distinct intersection points, with the burden being higher in men than in women during the period 1992–2006. This may be attributed to the real estate development boom that followed the Deng Xiaoping’s Southern Tour Speeches in 1992, leading to greater male participation in industrial construction activities [35], which increased the mechanical load on the joints of the hand. After 2007, as China’s economic structure gradually shifted towards the tertiary sector, female labor participation increased in areas such as office work and manual operations [36]. Repetitive hand movements in these roles likely contributed to a progressively higher burden of hand OA among women. Additionally, women inherently have a higher incidence of hand-related conditions such as tenosynovitis [37], which may further exacerbate the burden of hand OA in this population.

Age-effect analysis revealed that the CYR for hand, hip, and other site OA was highest in the population aged 95 years and above, whereas the CYR for knee OA peaked in the 80–84 age group. The earlier peak for knee OA may be related to biomechanical factors, as the knee bears substantial weight and is susceptible to earlier degenerative changes [8]. Period-effect analysis further validated the overall temporal trends, indicating a consistent increase in the YLDs burden for all OA sites over time, with the CYR peaking universally during the 2019–2023 period. This suggested that the impact of overarching risk factors – such as population aging and rising obesity rates – on the OA burden has become increasingly pronounced in recent years [38]. Cohort-effect analysis found that within the same age group, the CYR was lower in earlier birth cohorts compared to later ones. This indicates that individuals born more recently face a higher burden of OA-related disability at the same age than those born earlier. Therefore, the burden of OA was influenced not only by individual aging but also by period-specific factors and birth cohort effects.

Projection analyses predicted that YLD counts attributable to OA across all anatomical sites will keep rising, indicating that Chinese older adults will continue to face a heavy and long-term OA burden. This underscores the urgent need for integrated prevention and management interventions. Integrating OA into the national chronic disease management system could be a pivotal step. Furthermore, strengthening obesity management, with a particular focus on female populations, is critically important. Randomized controlled trials have confirmed that weight loss achieved via regular physical exercise and a balanced diet can effectively relieve OA symptoms [39]. Although age serves as a non-modifiable risk factor for site-specific OA, the popularization of OA screening tools adapted to community and primary healthcare settings facilitates the early and standardized diagnosis and treatment of elderly patients. Meanwhile, targeted health education regarding the rational use of electronic devices should be launched for elderly adults to promote ergonomic smartphone usage habits. In addition, the development of innovative OA therapeutics, including targeted gene therapy and nano-targeted drug delivery systems, is indispensable for improving OA management [40], 41].

This research has several limitations. First, the GBD 2023 estimates rely on ICD-10 codes to identify OA cases, which primarily capture diagnosed and often more severe cases. Consequently, individuals with OA who have not sought medical care are likely missed, resulting in a probable underestimation of the true OA burden. Second, the GBD 2023 methodology employs modeling to generate estimates, which inherently introduces potential biases and may not fully reflect the actual situation. Third, this study relied on aggregated GBD estimation data and lacked individual-level information regarding OA diagnosis status, exposure factors, and covariates, making it impossible to evaluate risk factors at the individual level. Fourth, the lack of data on OA severity across different provinces in China limits the ability to adequately account for regional variations, thereby constraining the development of more precisely targeted OA prevention and control strategies.

## Conclusions

From 1990 to 2023, the overall YLD burden of site-specific OA rose among Chinese elderly adults, with hand OA showing the largest growth rate and knee OA bearing the heaviest burden. Obvious gender and age disparities existed across all OA subtypes by anatomical site. YLD counts for OA at all sites are projected to keep rising from 2024 to 2035, indicating sustained challenges for the prevention and treatment of OA among Chinese elderly populations. This study adds complementary evidence for targeted, precise prevention, and control strategies tailored to specific anatomical sites, genders, and age groups, and highlights the urgent need to prioritize OA prevention and management within national chronic disease control programs. Future research should integrate domestic Chinese cohorts, disease registries or hospital databases to conduct individual-level analyses and further identify risk factors for OA incidence and progression. From a practical perspective, primary care and community training programs should strengthen health education covering early OA screening, weight management, joint protection, physical activity guidance, and rational use of electronic devices.

## Supplementary Material

Supplementary Material

Supplementary Material

Supplementary Material

Supplementary Material

Supplementary Material

Supplementary Material

Supplementary Material

Supplementary Material

Supplementary Material

Supplementary Material

Supplementary Material
