# Supplementary material for: Trends in disability burden of site-specific osteoarthritis among elderly adults in China from 1990 to 2023 and projections to 2035: a comprehensive analysis based on GBD 2023
Source: Open Med (Wars). 2026 Nov 12;21(1):20261519. doi: 10.1515/med-2026-1519 (PMC13758589; doi:10.1515/med-2026-1519)
Supplement: Supplementary file 1 [file j_med-2026-1519_suppl_007.pdf]

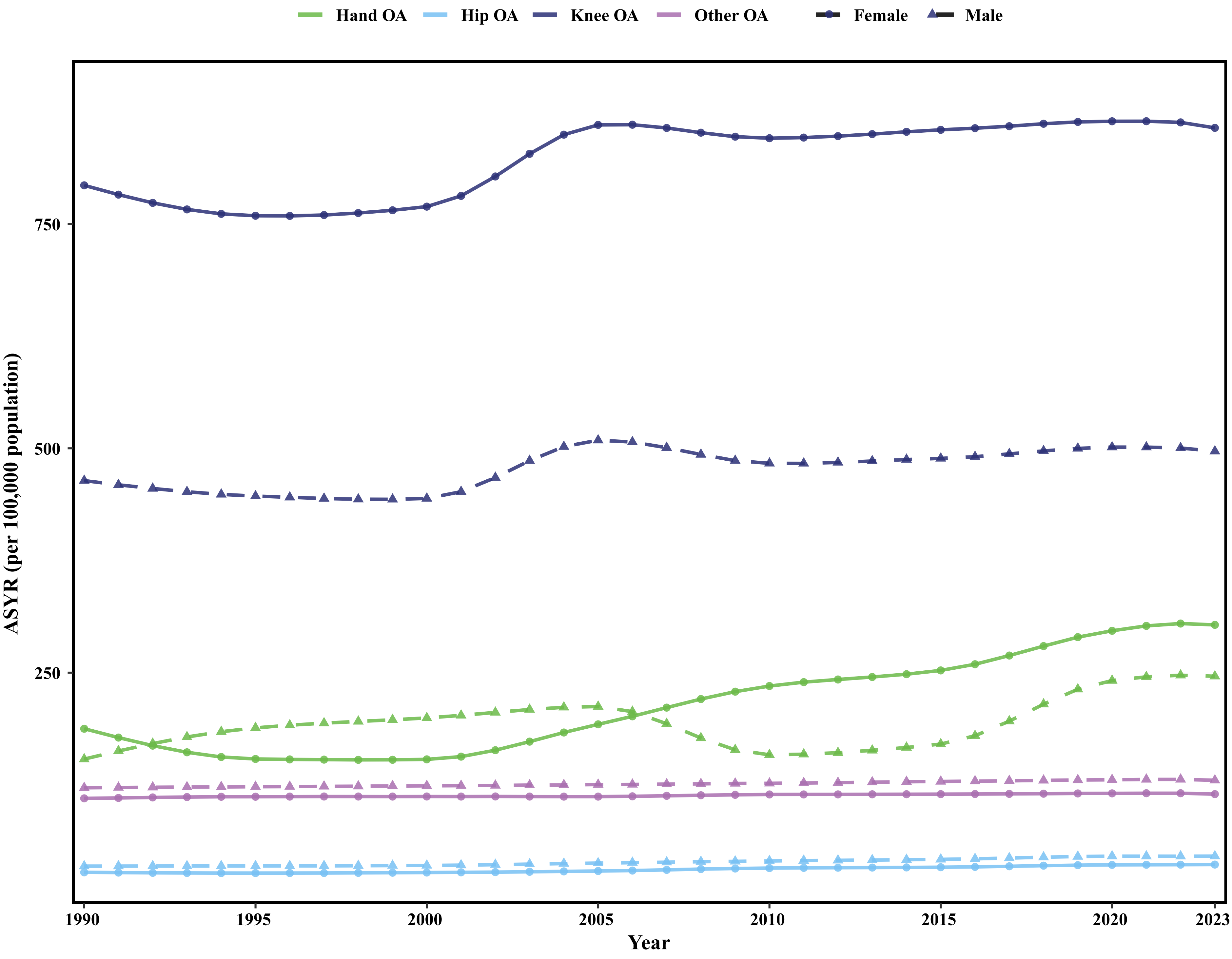
Hand OA
Hip OA
Knee OA
Other OA
Female
Male
ASYR (per 100,000 population)
750
500
250
1990
1995
2000
2005
2010
2015
2020
2023
Year
