# Supplementary material for: Trends in disability burden of site-specific osteoarthritis among elderly adults in China from 1990 to 2023 and projections to 2035: a comprehensive analysis based on GBD 2023
Source: Open Med (Wars). 2026 Nov 12;21(1):20261519. doi: 10.1515/med-2026-1519 (PMC13758589; doi:10.1515/med-2026-1519)
Supplement: Supplementary file 3 [file j_med-2026-1519_suppl_009.pdf]

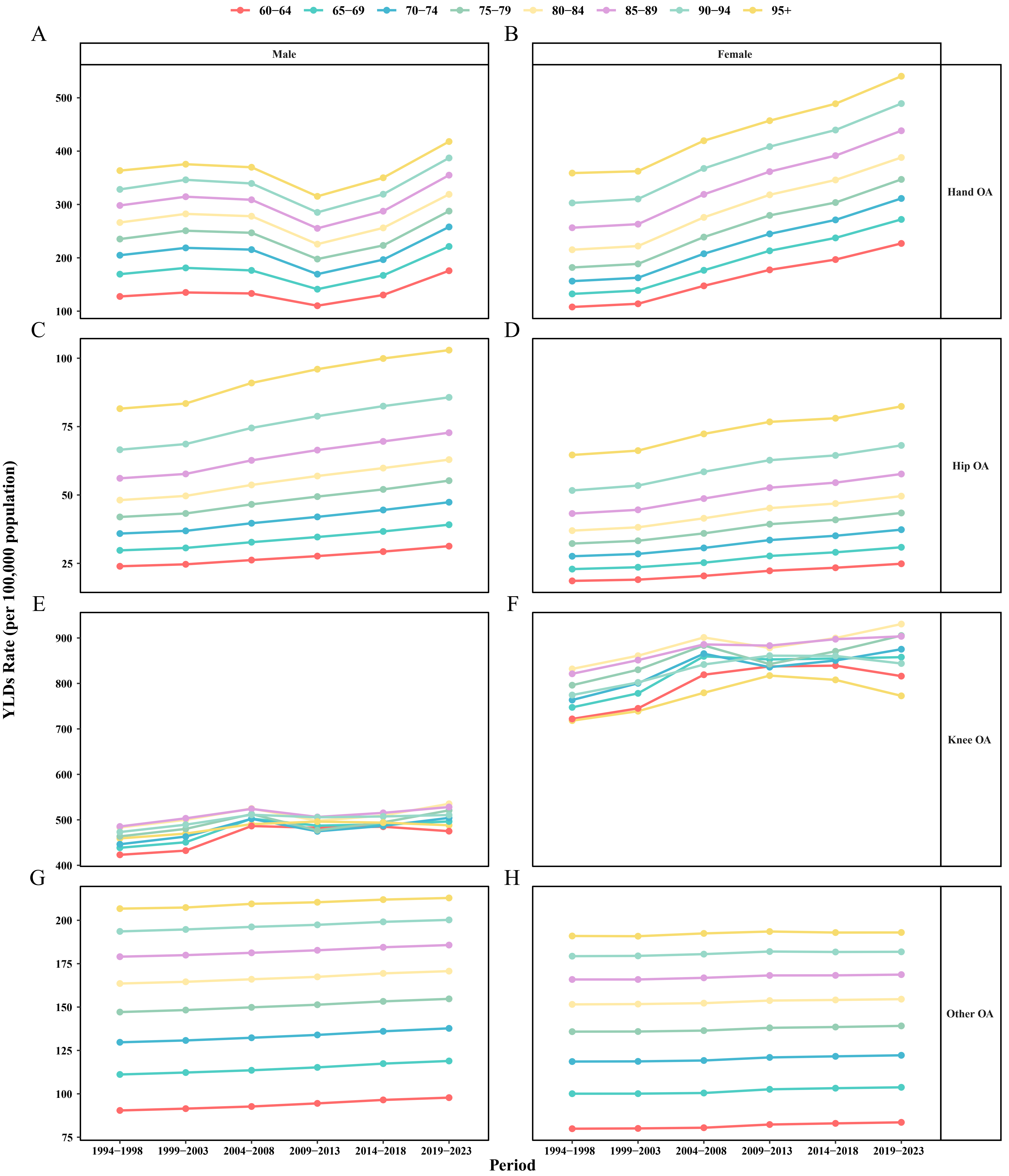

60−64
65−69
70−74
75−79
80−84
85−89
90−94
95+
A
B
C
D
E
F
G
H
Male
Female
Hand OA
Hip OA
Knee OA
Other OA
YLDs Rate (per 100,000 population)
Period
1994−1998
1999−2003
2004−2008
2009−2013
2014−2018
2019−2023
