# Supplementary material for: Trends in disability burden of site-specific osteoarthritis among elderly adults in China from 1990 to 2023 and projections to 2035: a comprehensive analysis based on GBD 2023
Source: Open Med (Wars). 2026 Nov 12;21(1):20261519. doi: 10.1515/med-2026-1519 (PMC13758589; doi:10.1515/med-2026-1519)
Supplement: Supplementary file 4 [file j_med-2026-1519_suppl_010.pdf]

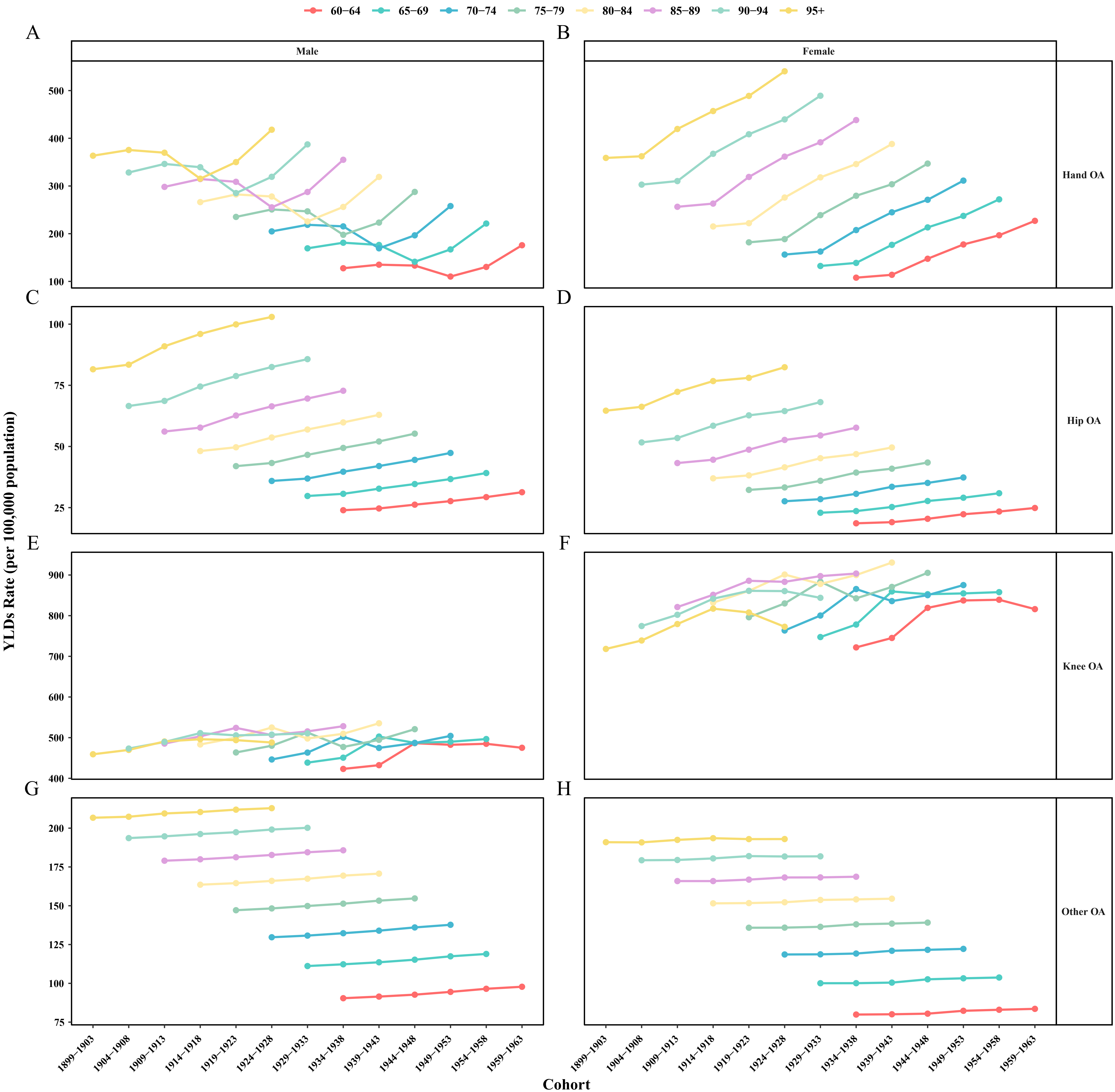

60−64
65−69
70−74
75−79
80−84
85−89
90−94
95+
A
B
C
D
E
F
G
H
Male
Female
Hand OA
Hip OA
Knee OA
Other OA
YLDs Rate (per 100,000 population)
Cohort
1899−1903
1904−1908
1909−1913
1914−1918
1919−1923
1924−1928
1929−1933
1934−1938
1939−1943
1944−1948
1949−1953
1954−1958
1959−1963
