# Supplementary material for: Trends in disability burden of site-specific osteoarthritis among elderly adults in China from 1990 to 2023 and projections to 2035: a comprehensive analysis based on GBD 2023
Source: Open Med (Wars). 2026 Nov 12;21(1):20261519. doi: 10.1515/med-2026-1519 (PMC13758589; doi:10.1515/med-2026-1519)
Supplement: Supplementary file 5 [file j_med-2026-1519_suppl_011.docx]

Figure S1 Trends in ASYR of site-specific OA among Chinese elderly adults by gender, 1990–2023. ASYR, age-standardized YLDs rate; YLDs, years lived with disability. Green lines denote hand OA, light blue lines denote hip OA, dark blue lines denote knee OA, and purple lines denote other-site OA. Solid lines represent females, and dashed lines represent males.

Figure S2 Age effect analysis of YLDs rates for hand and knee OA among Chinese elderly adults by gender.

(A, B) Hand OA; (C, D) Hip OA; (E, F) Knee OA; (G, H) Other OA. OA, osteoarthritis; YLDs, years lived with disability. Male data are displayed on the left panels, and female data on the right panels; different colors indicate distinct periods.

Figure S3 Period effect analysis of YLDs rates for hand and knee OA among Chinese elderly adults by gender.

(A, B) Hand OA; (C, D) Hip OA; (E, F) Knee OA; (G, H) Other OA. OA, osteoarthritis; YLDs, years lived with disability. Male data are displayed on the left panels, and female data on the right panels; different colors indicate distinct age groups.

Figure S4 Cohort effect analysis of YLDs rates for hand and knee OA among Chinese elderly adults by gender.

(A, B) Hand OA; (C, D) Hip OA; (E, F) Knee OA; (G, H) Other OA. OA, osteoarthritis; YLDs, years lived with disability. Male data are displayed on the left panels, and female data on the right panels; different colors indicate distinct age groups.

Supplementary Tables

**Table S1.** Number of YLDs and ASYR for site-specific OA among elderly adults in China, 1990-2023.

**Table S2.** Joinpoint regression analysis of the ASYR for site-specific OA among elderly adults in China, 1990-2023.

**Table S3.** Number of YLDs and ASYR for site-specific OA among elderly adults in China by gender, 1990-2023.

**Table S4.** Age and period effects on the YLDs rate for site-specific OA among elderly adults in China by gender.

**Table S5.** Cohort effects on the YLDs rate for site-specific OA among elderly adults in China by gender.

**Table S6.** Projected number of YLDs and ASYR for site-specific OA among elderly adults in China by gender, 2024-2035.
